# Diversity, resistance and vector competence of endophilic anophelines from southern Ghana

**DOI:** 10.5281/zenodo.10876351

**Published:** 2015-10-31

**Authors:** Michael Osae, Alessi Kwawukume, Michael Wilson, David Wilson, Lizette L. Koekemoer

**Affiliations:** 1 Graduate School of Nuclear and Allied Sciences, University of Ghana, Atomic Campus, Kwabenya, Accra, Ghana.; 2 Radiation Entomology and Pest Management Centre, Biotechnology and Nuclear Agriculture Research Institute, Ghana Atomic Energy Commission, Kwabenya, Accra, Ghana.; 3 WITS Research Institute for Malaria, Faculty of Health Sciences, University of the Witwatersrand and the Vector Control Reference Laboratory, National Institute for Communicable Diseases, Sandringham, Johannesburg, South Africa.; 4 Noguchi Memorial Institute for Medical Research, University of Ghana, Legon, Accra, Ghana.; 5 Department of Animal Biology and Conservation Sciences, University of Ghana, Legon, Accra, Ghana.

## Abstract

**Background:**

As part of efforts to monitor the impact of vector control strategies so that they can be improved and more targeted, we collected baseline data on aspects of the bionomics of endophilic anophelines in southern Ghana.

**Materials and Methods:**

Indoor resting anophelines were collected using mouth aspirators and pyrethroid spray catch. *Anopheles* females were identified to species level using morphological characteristics and sibling species were distinguished by PCR. The presence of the L1014F mutation, conferring resistance to insecticides, was determined in *An. gambiae s.s.* and *An. coluzzii* samples using TaqMan real-time PCR. Host blood meal sources were determined by PCR, and the presence of *Plasmodium falciparum* circumsporozoite proteins determined by ELISA.

**Results:**

A total of 892 female *Anopheles* (31% *An. gambiae*, 41% *An. coluzzii* and 28% *An. funestus*) were collected from six villages. The L1014F mutation was almost fixed in all populations studied (allele frequencies: 0.87-1.00). Both *An. gambiae s.l.* and *An. funestus* fed mainly on humans, with a human blood index of 1, although some animal feeding was recorded in *An. gambiae*. *P. falciparum* was detected in all ecological zones and in all three major vector species, being 4.9% in *An. funestus*, 3.8% in *An. gambiae s.s.* and 1.1% in *An. coluzzii*.

**Conclusions:**

These findings suggest that the three major vectors of malaria are present in all ecological zones of southern Ghana and contribute to disease transmission. The near fixation of the L1014F mutation in southern Ghana poses a great threat to vector control, thus highlighting the urgent need to implement measures to maintain the efficacy of current control tools and to develop novel control strategies.

## 1 Introduction

In Ghana, four dominant malaria vector species are responsible for malaria transmission: *Anopheles funestus s.s.*, and three members of the *An. gambiae* complex: *An. gambiae s.s.*, *An. arabiensis* and *An. coluzzii* [1]. *An. gambiae s.l.* and members of the *An. funestus* group are sympatric over much of their range in Africa [2]. Studies in southern Ghana have found that *An. gambiae s.s.*, *An. coluzzii* and *An. funestus* are the dominant malaria vector species [3-6]. These species are sympatric across much of Ghana but *An. gambiae s.s.* predominates in the middle belt, characterised by forest type vegetation, whereas *An. coluzzii* predominates in the northern and coastal savannah regions [7,8]. However, this information alone is too general and cannot be used to extrapolate effective vector control strategies against these species under the diverse ecological conditions experienced in Ghana. It has been established that occurrence, population density and vector competence of *An. gambiae s.l*. and *An. funestus* vary geographically and are influenced by a number of environmental conditions, such as climate, rainfall and vegetation, as well as activities which influence their breeding [2,3,7,9,10]. It is therefore essential to understand the distribution and population dynamics of these anophelines in the various ecological settings before implementing appropriate malaria vector control interventions.

In Ghana, the national vector control programme, spearheaded by the Ghana National Malaria Control Program (NMCP), among other malaria control initiatives, prioritises use of insecticide-treated bednets (ITNs) and indoor residual spraying (IRS) as the main malaria vector control tools [1]. These strategies rely heavily on few insecticides recommended by WHO [11]. Unfortunately, there is strong evidence that mosquito populations in Ghana have developed resistance to insecticides used in malaria vector control [4-8,12]. This development calls for a shift towards integrated vector management (IVM). There are several approaches available, including, but not limited to: insecticide-treated wall linings (ITWL), entomopathogenic fungi, larviciding, and the development and implementation of novel strategies to counter this problem. In addition, insecticide resistance management needs to be implemented to prolong effectiveness of the insecticides currently available for malaria vector control [1]. It is against this background that the studies reported in this article focused on understanding the bionomics and susceptibility levels of vector populations from southern Ghana as an essential step towards the development and implementation of effective IVM with a strong insecticide resistance management component.

## 2 Materials and methods

### 2.1 Study area

**Figure 1.**
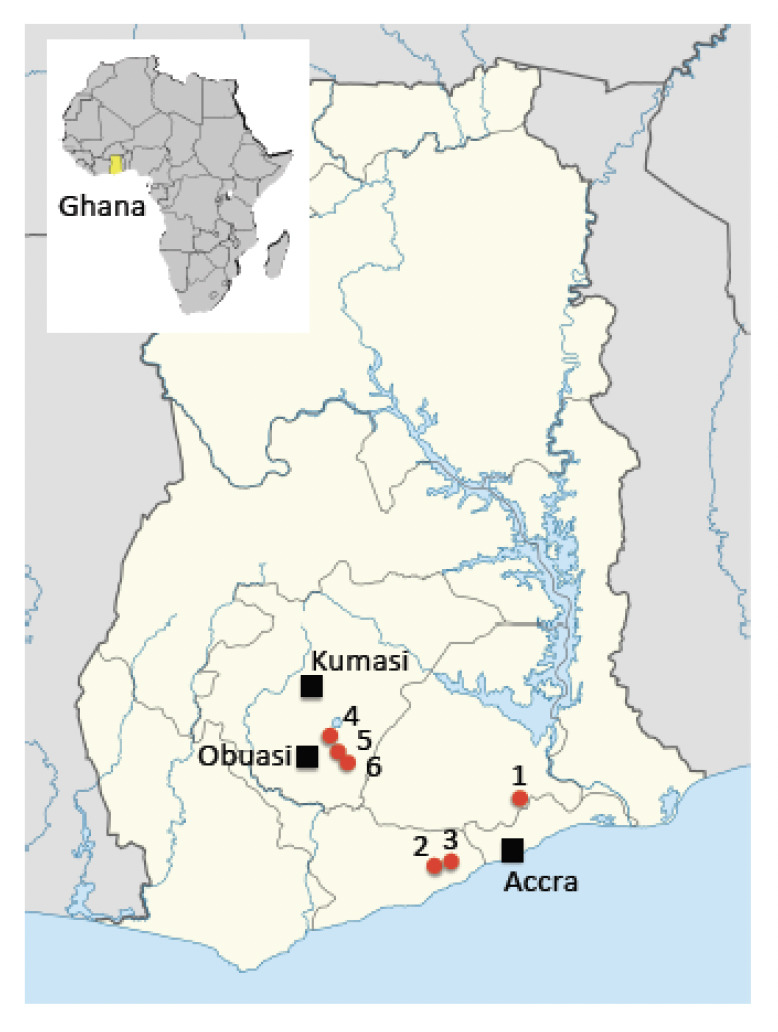
Map of Ghana showing the geographical location of study villages. 1: Osorongma, 2: Okyereko, 3: Adawukwa, 4: Kusa Dinkyeae, 5: Atatam, 6: Agyenkwaso.

The study was carried out in six villages in southern Ghana, covering three ecological zones: forest ecological zone (FEZ), forest transition ecological zone (FTEZ) and coastal savannah ecological zone (CSEZ) (Figure 1).

In the FEZ, mosquitoes were collected from three villages: Atatam (06^°^ 17.377”N, 001o 27.545”W), Agyenkwaso (06^°^ 15.547”N, 001^°^ 26.689”W) and Kusa Dinkyeae (06^°^ 18.894”N, 001^°^ 29.622”W), all located in the Adansi North District of the Ashanti Region (Figure 1). Atatam lies 257 m above sea level, is surrounded by mountains, from which several streams reach the village, and has 1,220 inhabitants. Agyenkwaso lies 223 m above sea level, with a population of about 1,300, while Kusa Dinkyeae has only 800 inhabitants and lies 268 m above sea level. Atatam and Agyenkwaso are proximal to each other while Kusa Dinhyeae is more isolated in the forest. A mountain separates Atatam and Kusa Dinkyeae. The main economic activity in these three villages is cocoa farming, interspersed with coffee, oil palm and subsistent farming. All three villages have been targeted by expansion of the Obuasi malaria control programme run by the AngloGold Ashanti Gold Mine because of their proximity to Obuasi municipality.

From the CSEZ, two villages, Gomoa Okyereko (05^°^ 24.828”N, 000^°^ 36.284”W) and Gomoa Adawukwa (05^°^ 24.960N 000o 36.729”), were sampled. Okyereko and Adawukwa are located in the Gomoa East District of the Central Region of Ghana, with an elevation of 19 and 22 meters above sea level, respectively. Okyereko is an irrigated rice farming village with about 1,500 inhabitants. Water from a dam about 500 m away from the village flows through canals to irrigate the rice fields, which are less than 100 m from the village. Flooded rice fields provide active breeding sites for different species of mosquitoes, including anophelines. Cement block houses with corrugated iron sheet roofing dominate, and eaves are either open or closed. Adawukwa is located about 500 m from Okyereko, and the two villages are separated by a river. About 500 m upstream the river, is a swamp where sugarcane cultivation, the main occupation of the inhabitants, takes place. There are about 1,200 people in the village, who, besides sugarcane farming, are involved in maize farming and sugarcane alcohol distillation. There is a brick and roofing tile factory in the village and, as a result, most of the houses, which are either made of brick or cement blocks, are roofed with brick tiles and often have open eaves. Apart from a few households with ITNs and the use by individuals of mosquito-repelling coils, there is no active malaria control programme running in any of the six villages. Domestic animals, such as goat, sheep, chicken, dogs, cats and pigs, are present in all villages. Cattle were not seen in any of the villages sampled.

The sixth village where mosquito sampling was carried out, Osorongma (05^°^ 52.757”N, 000^°^ 06.563”W), lies 94 m above sea level and is located in the FTEZ. It is a peri-urban village close to Dodowa in the Dangme West District of the Greater Accra Region of Ghana, with about 200 inhabitants, who are mainly farming cassava and mango. Houses in the village are a mix of cement block and mud houses, all with corrugated iron sheet roofing, usually with open eaves.

### 2.2 Mosquito collections and field processing

Sampling was carried out between September 2011 and January 2012, spanning the minor rainy season (September - November) and the dry season (December - March). Prior to mosquito sampling, permission to gain access to each village was sought from an opinion leader, such as the chief or assemblyman. In all cases, verbal informed consent was obtained from the head of each household before mosquito collections were carried out. Mosquitoes were collected early in the morning between 6 and 10 am inside houses, using standard indoor resting collection methods (mouth aspirators and pyrethroid spray collection). Immediately after collection, mosquitoes were killed in a killing bottle containing ethyl ether, individually placed in 1.5 ml microcentrifuge tubes containing silica gel and labelled. All specimens were transported to the laboratory where they were morphologically identified and sorted into species groups using the keys by Gillies and Coetzee [13], and stored at −20**°**C for further processing.

### 2.3 Laboratory analysis

A sub-sample of 30 or all (where less than 30 mosquitoes were collected) for each round of collection from every village was selected randomly for molecular assays. The head and thorax of each specimen was dissected and placed in labelled microcentrifuge tubes for enzyme-linked immunosorbent assay (ELISA) to determine the presence of *P. falciparum* sporozoites. DNA from the rest of the specimen (legs, wings and abdomen) was extracted using standard procedures described by Collins *et al*. [14]. The extracted DNA was used for species identification, *kdr* mutation determination and blood meal analysis by polymerase chain reaction (PCR).

Specimens identified morphologically as *An. gambiae s.l*. were identified to species level using the PCR assay described by Scott *et al*. [15]. Those specimens identified as *An. gambiae s.l*. were further identified as *An. gambiae s.s.* or *An. coluzzii* using the method described by Favia *et al*. [16]. Specimens identified morphologically as *An. funestus* were identified to species-specific level using the PCR assays of Koekemoer *et al*. [17], and those that failed to amplify after a single repeat were subjected to the IGS-based real-time PCR protocol for identification of *An. funestus* subgroup developed by Vezenegho *et al*. [18].

The West African knockdown resistance mutation (L1014F) was assayed in samples identified as *An. gambiae s.s*. and *An. coluzzii* using the TaqMan real time PCR (RT-PCR) assay described by Bass *et al*. [19]. The presence of *P. falciparum* parasites was determined in all specimens identified to the species-specific level using the ELISA protocol of Wirtz *et al*. [20]. To determine the blood meal source in blood-fed mosquitoes, the multiplex PCR method developed by Kent and Norris [21] was used. The assay was used to test for the presence of human, cow, pig, goat or dog blood in the specimens. Any specimens that gave multiple blood meals were repeated for confirmation. To confirm blood meal type, PCR products for each blood type were sequenced and checked for alignment with the respective host blood sequence.

### 2.4 Data analysis

Mosquito species density was calculated as the number of each species divided by the total number of mosquitoes collected per room per collection date. Knockdown resistance (*kdr*) data were analysed using a Chi-square test for goodness of fit, from which the allele frequencies were generated. Human blood index (HBI) was calculated as number of specimen positive for human blood divided by the total number of specimens tested. Sporozoite rate was calculated as number of specimen positive for *P. falciparum* divided by total number tested.

Data for indoor resting density and *kdr* allele frequencies for each species were compared across villages using ANOVA and Tukey’s test used for means separation. Student's *t*-test was used to compare percentage prevalence of *An. gambiae s.s.* and *An. coluzzii* in each village. All statistical analyses were performed using SPSS version 16, except for Chi-square tests, which were performed online using the Hardy-Weinberg equilibrium calculator [22].

### 2.5 Ethical clearance

This study was exempt from ethical approval by the Institutional Review Board of the Noguchi Memorial Institute for Medical Research, University of Ghana on the basis that it did not involve human participants.

## 3 Results

### 3.1 Indoor resting mosquito density

A total of 925 indoor resting mosquitoes, comprising 640 (68%) female *An. gambiae s.l.*, 252 (27%) female *An. funestus*, 34 (4%) male anophelines and 9 (1%) *Culex spp.* were collected from the six villages (Table 1). The indoor resting densities of *An. gambiae s.l.* were generally higher (*P*<0.05) than those of *An. funestus* in all villages except Atatam and Agyenkwaso, where there was no significant differences in their abundance (*P*>0.05).

**Table 1.** Indoor resting mosquito densities in six villages in southern Ghana.

	Mean number of mosquitoes/room ± S.E.			
Village	*An. gambiae s.l.*	*An. Funestus* group	*Culex spp.*	Total
Atatam	7.20 ± 1.59^b^	7.45 ± 1.46^b^	0.00 ± 0.00^c^	14.65 ± 1.90^a^
Agyenkwaso	4.36 ± 1.15^b^	5.18 ± 1.33^ab^	0.27 ± 0.14^c^	9.81 ± 1.08^a^
Kusa Dinkyeae	8.63 ± 2.82^a^	1.50 ± 0.76^b^	0.00 ± 0.00^c^	10.13 ± 2.57^a^
Osorongma	10.00 ± 2.49^a^	0.00 ± 0.00^b^	0.00 ± 0.00^b^	10.00 ± 2.49^a^
Adawukwa	14.75 ± 2.60^a^	4.38 ± 2.78^b^	0.38 ± 0.38^c^	19.51 ± 1.15^a^
Okyereko	12.13 ± 2.19^a^	0.13 ± 0.13^b^	0.33 ± 0.33^b^	12.39 ± 2.13^a^
Total	9.18 ± 0.91^b^	3.54 ± 0.65^c^	0.11 ± 0.05^d^	12.83 ± 0.90^a^

Means in the same row with different letters are significantly different at P<0.05. S.E.: standard error of mean*.*

From a total of 308 female *An. gambiae s.l.* processed, 176 (57.71%) were identified as *An. coluzzii* and 132 (42.86%) as *An. gambiae s.s.* (Table 2). *An. gambiae s.s.* was predominant in Atatam, Agyenkwaso, and Osorongma, and was the only member of the species complex collected from Kusa Dinkyeae. On the other hand, collections from Adawukwa and Okyereko, which are very close to each other, were exclusively *An. coluzzii*.

**Table 2.** Relative prevalence (%) of *An. gambiae s.s*. and *An. coluzzii* in six villages in southern Ghana.

Village	N	*An. coluzzii*	*An. gambiae s.s.*
Atatam	47	1.96 ± 1.96^b^	98.04 ± 1.96^a^
Agyenkwaso	22	23.61 ± 1.39^b^	76.39 ± 1.39^a^
Kusa Dinkyeae	29	0.00^b^	100.00^a^
Osorongma	43	6.00 ± 3.24^b^	94.00 ± 3.24^a^
Adawukwa	79	100.00^a^	0.00^b^
Okyereko	88	100.00^a^	0.00^b^
Total	308	57.71 ± 11.76^a^	42.86 ± 11.69^a^

Means in the same row with different letters are significantly different at P<0.05. S.E.: standard error of mean.

### 3.2 Prevalence of *kdr* mutations

The L1014F mutation was present in both *An. gambiae s.s.* and *An. coluzzii* (Table 3). The allele frequencies were slightly lower in *An. coluzzii*, ranging from 0.75 in the Osorongma population to 0.90 in the Agyenkwaso population. In Adawukwa and Okyereko, the allele frequencies were 0.87 and 0.86, respectively. In *An. gambiae s.s.*, it was almost fixed in all the populations, being 0.97 in Atatam, 0.99 in Osorongma and 1.00 in Agyenkwaso and Kusa Dinkyeae.

**Table 3.** Allele distribution of the west African *kdr* mutation in *An. gambiae s.l.* from six villages in southern Ghana.

	*kdr* Allele frequencies (n)	
Village	*An. coluzzii*	*An. gambiae s.s.*
Atatam	−*	0.97 (48)^a^
Agyenkwaso	0.90 (5)^a^	1.00 (17)^a^
Kusa Dinkyeae	−	1.00 (29)^a^
Osorongma	0.75 (2)a	0.99 (39)^a^
Adawukwa	0.87 (79)^a^	−
Okyereko	0.86 (88)^a^	−

* Insufficient specimens available for analysis. Values in the same column with same superscript letters are not significantly different at P<0.05.

### 3.3 Blood meal source and sporozoite rates

Results for blood meal source identification are shown in Table 4. The PCR method could not identify the source of blood meal in some specimens despite physical evidence of blood in the abdomen. A total of 414 specimens were assayed, with success rates ranging from 50 to 100%. A high level of anthropophagy was recorded for the three endophilic anophelines in all villages sampled. *An. funestus* populations from the three ecological zones sampled were exclusively anthropophagic (HBI = 1), i.e. no animal feeding was observed. *An. gambiae s.s.* and *An. coluzzii* populations were similarly highly anthropophagic; however, evidence of animal blood feeding was recorded in both species. All *An. gambiae s.s.* and *An. coluzzii* specimens that fed on animals had also fed on humans, except for one *An. coluzzii* specimen from Okyereko that had exclusively fed on animal blood (cow).

**Table 4.** Blood meal source for three endophilic anophelines from six villages in southern Ghana.

	Blood Meal Source (%)					
Species	N	NIS (%)	Human	Animal	Mixed	HBI
*An. gambiae s.s.*	128	89 (70)	89 (100)	10 (11)	10(11)	1
*An. coluzzii*	166	115 (60)	114 (99)	4 (3)	3 (3)	0.99
*An. funestus*	120	101 (84)	101 (100)	0 (0)	0 (0)	1
Total	414	305 (74)	304 (99.7)	14 (5)	13 (5)	0.99

NIS: Number identified successfully. HBI: human blood index. Percentages in blood meal source column may not add up to 100, as some specimens contained mixed blood meals.

*Plasmodium falciparum* infection rates were determined for 429 specimens, comprising 131 *An. gambiae s.s.*, 175 *An. coluzzii* and 123 *An. funestus* (Table 5). There was evidence of infection in all the villages except Okyereko, where *P. falciparum* was not present in any of the 90 specimens processed. After pooling the data from all villages, infectivity was highest in *An. funestus,* followed by *An. gambiae s.s*. and lowest in *An. coluzzii*.

**Table 5.** *Plasmodium falciparum* sporozoite infection rates of indoor resting anophelines from six villages in southern Ghana

	% Infected (n)		
Village	*An. funestus*	*An. coluzzii An.*	*gambiae s.s.*
Atatam	4.76 (63)	0 (1)	2.17 (46)
Agyenkwaso	2.86 (35)	20.00 (5)	11.76 (17)
Kusa Dinkyeae	8.33 (12)	-	0 (29)
Osorongma	0 (1)	0 (3)	5.13 (39)
*Adawukwa*	10 (10)	1.27 (79)	-
Okyereko	0 (2)	0 (88)	-
Total	4.88 (123)	1.14 (176)	3.8 (131)

## 4 Discussion

### 4.1 Indoor resting mosquito diversity

*Anopheles gambiae s.l.* and *An. funestus s.s.* were the only anophelines collected from the villages sampled. The in-door resting densities of these species from the FEZ did not differ, except for Kusa Dinkyeae where the indoor resting density of *An. gambiae s.l*. was higher than that of *An. funestus*. By contrast, more *An. gambiae s.l*. were collected indoors compared to *An. funestus* in Adawukwa and Okyereko from the CSEZ. Only one *An. funestus* specimen was collected from Osorongma in the FTEZ. Members of the *An. gambiae* complex showed varied distribution across the study area, with collections from the FEZ and FTEZ being predominantly *An. gambiae s.s*. with very few *An. coluzzii*, except in Kusa Dinkyeae, where no *An. coluzzii* was collected. *An. gambiae s.l*. from the CSEZ were, however, exclusively *An. coluzzii*. These results are similar to other findings from southern Ghana [4,6,7]. All collections from Obuasi in 2011 [6], a location closest to the three FEZ sampling sites, were *An. gambiae s.s*. Similarly, 100% of all collections from Kumasi, a locality in the FEZ, were *An. gambiae s.s.*, and 98% from Osorongma in the FTEZ [4]. Results from Okyereko and Adawukwa (100% *An. coluzzii* from each site) also confirm findings dating back to 2004 [4]. The relative abundance of these two species, which until recently were considered molecular forms of the same species (*An. gambiae s.s.*), has been associated with breeding site characteristics. *An. coluzzii* tends to be associated with flooded or irrigated sites that provide permanent breeding conditions, whereas *An. gambiae s.s*. is associated with rain-dependent temporary sites [23].

The results obtained in the current study are in accordance with several reports across Africa, where the three species occur in sympatry over much of their range [2,24]. Prevalence of anophelines in any locality is influenced by ecological factors, such as availability of preferred hosts, breeding sites and environmental variables, such as temperature and relative humidity [7,25,26]. In this study, mosquitoes were collected using indoor collection techniques; therefore, availability of host and resting behaviour were the main expected determinants of occurrence. As all species collected in this study are highly anthropophilic, endophagic and endophilic [2], it was expected that they would be equally represented in the collections. However, this was not the case. The differences in the distribution observed could be attributed to the availability of breeding sites around the villages where mosquitoes were sampled. Presence of breeding sites suitable for proliferation of *An. gambiae s.s*. and *An. funestus*, i.e. availability of temporary water puddles during the rainy season due to poorly permeable clay soils and several rivers around the villages of Atatam and Agyenkwaso, could be the underlying factors for the presence of both species in equal densities. Since there were no permanent water bodies in Kusa Dinkyeae this may explain the predominance of *An. gambiae s.s*.

The breeding sites around villages in the CSEZ are different from those in the FEZ. Okyereko is an irrigated rice-cultivating community, and Adawukwa has a vast swamp and a nearby stream. The rice fields close to these two villages are ideal breeding sites for *An. coluzzii* and, to a lesser extent, *An. funestus*, while the edges of the stream and swamps are ideal sites for *An. funestus* breeding. This probably explains the presence of both species in Okyereko and Adawukwa. The highest density of *An. funestus* was recorded in Adawukwa, which is located closer to a swamp, suitable for *An. funestus* breeding. Our findings agree with those by Charlwood and Edoh [26], who reported that anopheline adult density is negatively correlated with the distance between larval habitats and houses. The complete absence of *An. funestus* in Osorongma could be explained by the absence of suitable breeding sites, as there are no permanent water bodies suitable for *An. funestus* breeding near this village. Furthermore, according to de Souza *et al*. [7], the distribution of the members of the *An. gambiae* complex appears to be driven by environmental factors, with *An. gambiae s.s.* more predominant in the forested areas characterised by lower mean daily temperatures, whereas *An. coluzzii* is predominant in the northern and coastal savannah areas, where mean daily temperatures are slightly higher.

The densities reported here may not represent the actual annual anopheline distribution in the study areas. Sampling was only limited to two seasons: the minor rainy season (September to November, 2011) and the dry season (December 2011 - January 2012). Species composition and abundance varies with time of year [6]. Yawson *et al*. [4] collected 58 *An. funestus* out of 341 specimens from 10 collections in Osorongma during the major rainy season (June to September), whereas in the present study, only one specimen of *An. funestus* was collected from the same village during the dry season. In this study, more *An. gambiae* was collected during the minor rainy season than the dry season and vice versa for *An. funestus* (data not shown).

### 4.2 Prevalence of the *kdr* mutation

We observed high variability in the prevalence of the L1014F mutation in *An. gambiae s.s.* and *An. coluzzii*. The near fixation of the L1014F mutation in the populations from the areas sampled is understandable, since this mutation is widespread and predominant in West Africa [27]. High frequencies of the L1014F mutation have been reported from same location or locations close to the sampled villages across southern Ghana [4-6,27]. Results from the current study show that the mutation is prevalent in both *An. coluzzii* (90%) and *An. gambiae s.s.* (98%). The L1014F mutation has shown a steady increase in *An. coluzzii* from different locations in southern Ghana since 2002 [4,8,28]. Around 2002-2004, the frequency was 1-3%, and increased to 54-79% by 2007/2008 [4,8,28]. The frequency recorded in this study for collections made in late 2011 and early 2012 ranged from 75 to 90% showing a further increase. This is not the first report of a temporal increase of the mutation in *An. coluzzii* in West Africa. Djegbe *et al*. [29] reported a near fixation of the allele in this species from Cotonou, and a significant temporal increase over a two-year period at Bohicon and Malanville in Benin. Santolamazza *et al*. [27] concluded that the distribution of the *kdr* mutation is non-uniform on the African continent and that its origin and spread in *An. gambiae s.l.* is an ongoing process.

The spread of the *kdr* mutation on the African continent has been attributed to introgression [30] and, to some extent, migration [29], driven mainly by widespread use of insecticides in agriculture and possibly for domestic purposes [31]. Insecticide usage is widespread in southern Ghana where this study was carried out [32]. In the forested areas where Atatam, Agyenkwaso and Kusa Dinkyeae are located, cocoa farming is the major agricultural activity, which has been and is still reliant on usage of insecticides of all classes, including organochlorines for capsids (*Distantiella theobroma* and *Sahlbergella singularis*) control [32]. Irrigated rice production around Okyereko and Adawukwa in the coastal savannah areas, and mango plantations of the eastern mango enclave of Ghana, around Osorongma, rely heavily on insecticide usage. Akogbeto *et al*. [33] demonstrated that insecticide-resistant *An. gambiae* populations are able to survive and proliferate in insecticide-contaminated soils and water from vegetable and cotton farms. Insecticide residues have been detected in water and soil samples from different parts of Ghana [32], suggesting the presence of heavy selection pressure on anopheline populations. Thus, whether the mutation enters the population by independent mutation events, introgression or by migration, it will easily be sustained and is likely to spread further in vector populations.

### 4.3 Vector competence

The success rate of the PCR method in identifying blood meal source was generally high (84% for *An. funestus*, 70% for *An. gambiae s.s.* and 60% for *An. coluzzii*), although it did not include primers for all the possible hosts found in the villages where mosquitoes were sampled, such as chicken, sheep, bats or rodents. It could be possible that specimens for which the blood meal source could not be determined had fed on such animals. It is important to note all possible hosts within a locality where samples are being collected and to include primers specific for as many hosts as possible in the PCR testing. Alternatively, a technique involving screening of mosquito blood meals with avian- and mammalian-specific primer pairs followed by sequencing could be used [34]. Analysis of the sequences can be used to determine the actual species each mosquito specimen fed on.

All three major vector species from southern Ghana show high levels of anthropophagy, with *An. funestus* being exclusively anthropophagic consistent with their feeding behaviour. According to Sinka *et al*. [2] *An. funestus* shows fairly consistent behaviour (generally anthropophilic and endophilic) throughout its range, whereas members of the *An. gambiae* complex appear to exhibit greater phenotypic plasticity and opportunism in blood feeding than commonly thought. In the present study, *An. gambiae s.s*. and *An. coluzzii* demonstrated low levels of animal feeding, but all except a single specimen that fed on animals also fed on humans. This could imply that, in the absence of humans, they will likely feed on animals, but given the chance, will supplement with human blood.

With the high HBI recorded for both species in the two ecological zones, the sporozoite rate should reflect the prevalence of the parasite within the populations. This is evident in the variable sporozoite rates recorded for the three species. Thus, the sporozoite rate only reflects the prevalence of parasites in the population and indicates the competence of the species as vectors. The sporozoite rates recorded in the current study fall within the range of those recorded by other workers [3,5,6,12,35] in Ghana, confirming these areas as malaria endemic. However, a caveat on these data is that the ELISA method is known to often give false positive results, especially for *P. falciparum* and for zoophilic vector species [36]. The highest sporozoite rate of 20% in this study was recorded from Agyenkwaso, where some zoophily was also observed. Furthermore, the small sample size could also affect the sporozoite rate as all the sites with high rates had small sample sizes. Nevertheless, all three vector species are actively transmitting malaria in the villages sampled.

## 5 Conclusions

*Anopheles gambiae s.s.*, *An. coluzzii* and *An. funestus* are the major indoor resting malaria vectors in all the three ecological zones where this study was carried out, but showing variations in their distribution. *An. gambiae s.s.* predominates in the forested areas, whereas *An. coluzzii* predominates in the coastal savannah and *An. funestus* shows a wider distribution across southern Ghana. This distribution is highly influenced by breeding site availability and environmental factors. Thus, any research and development activity that focuses on developing control tools targeting these major malaria vectors might have to be carried out across all the ecological zones to be relevant for area-wide implementation. Similarly, to carry out any control programme across these ecological zones, the tools must target all three species.

The L1014F *kdr* mutation is present at very high frequencies in all three ecological zones, with its spread in the population nearing fixation in both *An. gambiae s.s*. and *An. coluzzii*. This implies that control programmes must include resistance management strategies and incorporate novel tools that are not affected by the *kdr* mutation. There should also be extensive resistance monitoring in the country and further research into the impact of this and other resistance mechanisms on vector control activities in Ghana.
